# Medical Student Experiences and Perceptions of ChatGPT and Artificial Intelligence: Cross-Sectional Study

**DOI:** 10.2196/51302

**Published:** 2023-12-22

**Authors:** Saif M I Alkhaaldi, Carl H Kassab, Zakia Dimassi, Leen Oyoun Alsoud, Maha Al Fahim, Cynthia Al Hageh, Halah Ibrahim

**Affiliations:** 1 Khalifa University College of Medicine and Health Sciences Abu Dhabi United Arab Emirates; 2 Department of Medical Science Khalifa University College of Medicine and Health Sciences Abu Dhabi United Arab Emirates; 3 Education Institute Sheikh Khalifa Medical City Abu Dhabi United Arab Emirates

**Keywords:** medical education, ChatGPT, artificial intelligence, large language models, LLMs, AI, medical student, medical students, cross-sectional study, training, technology, medicine, health care professionals, risk, technology, education

## Abstract

**Background:**

Artificial intelligence (AI) has the potential to revolutionize the way medicine is learned, taught, and practiced, and medical education must prepare learners for these inevitable changes. Academic medicine has, however, been slow to embrace recent AI advances. Since its launch in November 2022, ChatGPT has emerged as a fast and user-friendly large language model that can assist health care professionals, medical educators, students, trainees, and patients. While many studies focus on the technology’s capabilities, potential, and risks, there is a gap in studying the perspective of end users.

**Objective:**

The aim of this study was to gauge the experiences and perspectives of graduating medical students on ChatGPT and AI in their training and future careers.

**Methods:**

A cross-sectional web-based survey of recently graduated medical students was conducted in an international academic medical center between May 5, 2023, and June 13, 2023. Descriptive statistics were used to tabulate variable frequencies.

**Results:**

Of 325 applicants to the residency programs, 265 completed the survey (an 81.5% response rate). The vast majority of respondents denied using ChatGPT in medical school, with 20.4% (n=54) using it to help complete written assessments and only 9.4% using the technology in their clinical work (n=25). More students planned to use it during residency, primarily for exploring new medical topics and research (n=168, 63.4%) and exam preparation (n=151, 57%). Male students were significantly more likely to believe that AI will improve diagnostic accuracy (n=47, 51.7% vs n=69, 39.7%; *P*=.001), reduce medical error (n=53, 58.2% vs n=71, 40.8%; *P*=.002), and improve patient care (n=60, 65.9% vs n=95, 54.6%; *P*=.007). Previous experience with AI was significantly associated with positive AI perception in terms of improving patient care, decreasing medical errors and misdiagnoses, and increasing the accuracy of diagnoses (*P*=.001, *P*<.001, *P*=.008, respectively).

**Conclusions:**

The surveyed medical students had minimal formal and informal experience with AI tools and limited perceptions of the potential uses of AI in health care but had overall positive views of ChatGPT and AI and were optimistic about the future of AI in medical education and health care. Structured curricula and formal policies and guidelines are needed to adequately prepare medical learners for the forthcoming integration of AI in medicine.

## Introduction

Innovation drives health care and health professional education forward. Yet medical education has historically been slow to embrace major change. For example, despite the availability of digital infrastructure and multiple online resources, many medical schools continue to rely on traditional lectures and hands-on experiential learning and have not incorporated the “flipped classroom” model or virtual reality simulations into the curriculum [1]. In recent years, health systems have been challenged by large-scale disruptions, with significant and wide-sweeping impacts on medical education. The COVID-19 pandemic forced an abrupt leap into the virtual learning environment, expediting the widespread use of technology-enhanced learning [2]. Concomitantly, the pandemic contributed to increased awareness of social and health disparities, spurring the implementation of diversity initiatives and social determinants of health curricula in medical schools and residency programs worldwide [3]. We are currently on the precipice of another transformational shift in health care and medical education. Artificial intelligence (AI) and AI-based large language models (LLMs), such as ChatGPT, have the potential to revolutionize the way medicine is learned, taught, and practiced.

Since its launch in November 2022, ChatGPT has emerged as a fast and user-friendly LLM that can assist health care professionals, medical educators, students/trainees, and patients [4-6]. It is capable of amalgamating and processing large amounts of data and has received passing scores equivalent to a third-year medical student on steps 1 and 2 of the United States Medical Licensing Exam (USMLE) [7]. Moreover, ChatGPT can be used as a testing tool, providing learners with logical explanations for incorrect responses and allowing them to gain knowledge [8]. ChatGPT can provide medical students and residents with personalized learning experiences in a safe setting tailored to their learning styles and needs and supported with immediate feedback [8]. Students and trainees can also have access to readily synthesized evidence-based information that they can use in academic writing [9] and clinical care and decision-making. This can contribute to better training and, ultimately, improved patient care [10,11].

The use of generative AI in medical education is not without controversy. Legal and ethical concerns include bias, copyright and privacy infringements, and overreliance on the technology with potential dehumanization in the learning process [12-14]. ChatGPT has also been found to provide incorrect or fabricated data or “hallucinate,” whereby its generated responses may appear plausible and convincing but are inaccurate or illogical [15-18].

The literature on AI and ChatGPT is growing rapidly and is primarily focused on this technology as a transformative innovation and its capabilities, possibilities, and risks. Many studies discuss ChatGPT’s potential to significantly impact teaching and learning, but there is no consensus on how to incorporate it into the medical curriculum. Arguably, transformative innovation goes beyond policy and curricular changes; it disrupts the status quo and challenges the medical education community to question previously held beliefs and practices [19]. As the adoption of technology into medical education progresses, it becomes important to understand medical students’ and residents’ perceptions, concerns, and expectations. This understanding can identify gaps in their knowledge and skills to help educators and policymakers design and implement effective educational interventions tailored to student needs [20]. Therefore, we conducted a study of recently graduated medical students in an international academic medical center to gauge their experiences and perspectives on the uses of ChatGPT and AI in their medical training and on their future careers in medicine.

## Methods

### Ethical Considerations

We conducted a cross-sectional web-based survey of medical students in the United Arab Emirates. The Sheikh Khalifa Medical City Institutional Review Board approved this study with a waiver of informed consent (RS-804). We used the Checklist for Reporting Results of Internet E-Surveys (CHERRIES) to guide our reporting [21] (Multimedia Appendix 1).

### Setting and Participants

Participants included medical student applicants to all residency training programs in an academic medical center in the United Arab Emirates. There are currently 2 models of undergraduate medical education in the United Arab Emirates, whereby most medical schools have undergraduate entry (following high school) and a 6-year curriculum and 1 school has postgraduate entry (after a bachelor’s degree) and a 4-year curriculum. Application to residency training is open to graduates from medical schools worldwide. Graduate medical education in the United Arab Emirates is competency based and models the US training structure, with similar resident roles and responsibilities [22].

### Study Development

The open survey instrument was developed after a comprehensive review of the literature on ChatGPT and AI in health care and medical education and iteratively revised by a panel of 5 medical educators and bioinformatics specialists. The Formsite (Vroman Systems, Inc) survey tool was used. Questions were in English and aimed to understand the students’ formal and informal experiences with ChatGPT and AI in medical school, expectations of using ChatGPT and AI in residency training, and overall perceptions of the impact of AI and LLMs on health care and their professional careers. The instrument was pilot-tested on 15 medical students for length and clarity with only minor changes made based on their comments. These responses were not included in the data analysis. The final version consisted of 42 questions divided into 4 sections (Multimedia Appendix 2). Each survey question only allowed for 1 response, which could be freely changed until survey completion and submission. Following basic demographic questions, participants were asked about prior experiences with LLMs and digitally enhanced education, anticipated use of LLMs in residency training, and overall perceptions of AI technology.

### Data Collection

Between May 5, 2023, and June 13, 2023, an administrator who was not involved in the residency recruitment process invited all medical student applicants on site visits of the hospital and its training programs to scan a QR code that directed them to the web-based survey. Once scanned, the survey could be completed at any time. The first page of the survey provided the description and purpose of the study and explained that it was anonymous and confidential. Participation was voluntary and no incentives were offered. No IP addresses were collected. Consent to participate in the study was indicated by the completion and submission of the survey.

### Data Analysis

Data were analyzed using R (version 4.2.2; R Foundation for Statistical Computing). Descriptive statistics were used to tabulate the frequency of the variables. Subgroup analysis was performed to determine the correlation between the demographics and the different variables, and significance was assessed using the chi-square test. Regression analysis was used to determine the association between age, gender, and previous experience with positive perception while controlling confounding variables. *P*<.05 indicated a significant difference between the variables.

## Results

Of 325 applicants to the residency programs, 265 completed the survey (for an 81.5% response rate). The demographic characteristics of the participants are represented in Table 1. The majority of participants (n=174, 65.7%) were women, which is consistent with the gender distribution in the region’s medical schools and residency programs [23]. Respondents trained in medical schools in multiple countries, but most participants graduated from local medical schools (n=187, 70.6%) and were applying to different medical specialties.

Respondents reported minimal incorporation of advanced technology into their medical school curricula (Table 2). The vast majority of respondents also denied using ChatGPT in medical school, with only 20.4% (n=54) using the technology to help complete written assessments and less than 10% (n=25, 9.4%) using ChatGPT to help write patient notes (Table 2). Despite their limited experience with ChatGPT, more students planned to use it during residency, primarily for exploring new medical topics and research (n=168, 63.4%) and for exam preparation (n=151, 57%). Less than half intended to use ChatGPT to write case reports (n=122, 46%) or research papers (n=127, 47.9%), and fewer than a third of respondents anticipated using ChatGPT for clinical purposes, such as writing patient notes (n=79, 29.8%) or assisting in decision-making (n=73, 27.5%) (Table 3).

**Figure 1:**
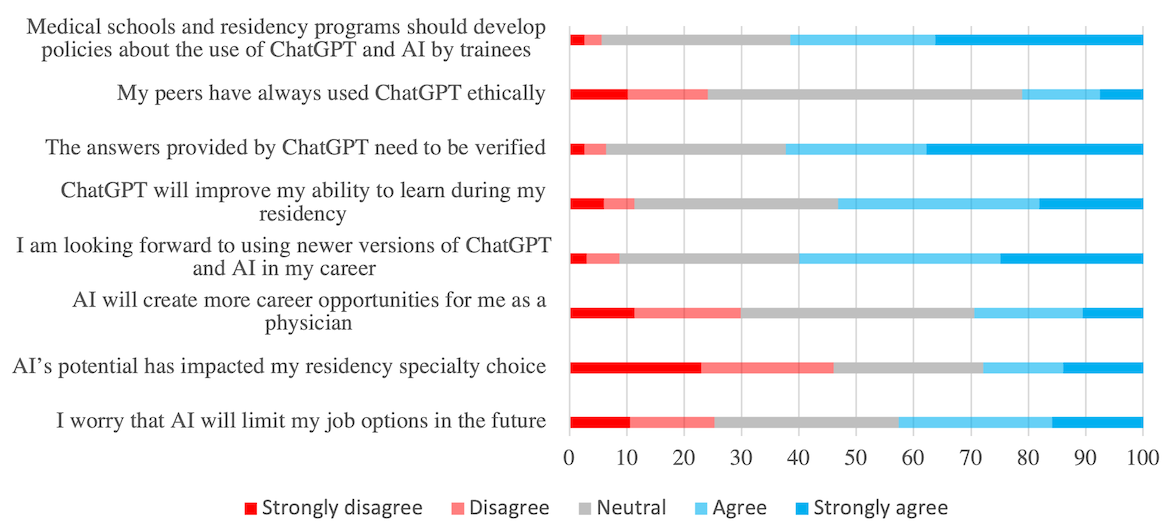
Perceptions of artificial intelligence (AI) for career and education.

**Figure 2:**
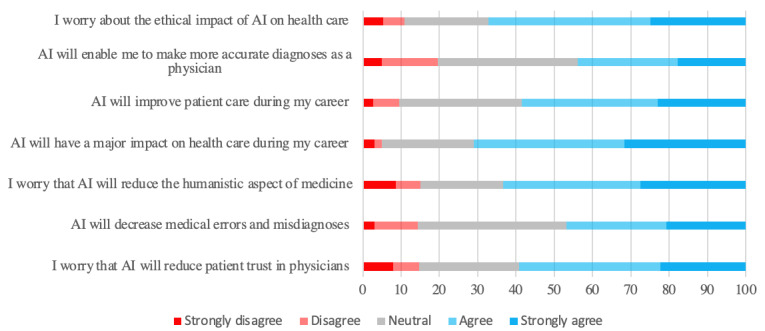
Perceptions of artificial intelligence (AI) for patient care.

Respondents expressed interest in using newer versions of ChatGPT (n=159, 60%) and believed that it would improve their learning (n=141, 53.2%). However, they were more ambivalent about its utility in career progression, with many students expressing uncertainty about AI’s impact on their future opportunities (n=108, 40.8%) and job options (n=85, 32.1%), whereas only 78 participants (n=29.4%) agreed that ChatGPT would expand career opportunities (Figure 1). Most respondents were optimistic about AI’s potential and agreed (n=188, 70.9%) that AI will have a major impact on health care during their careers by improving patient care (n=155, 58.5%), though less than half believed that it would improve diagnostic accuracy (n=116, 43.8%) or reduce medical errors (n=124, 46.8%). Although few students frankly disagreed with the positive impact of AI on clinical care, many responses were neutral (Figure 2).

Concerning the ethical implications of AI, the students believed that AI could decrease humanism in medicine (n=168, 63.4%) and reduce patient trust in physicians (n=157, 59.2%) (Figure 2). The majority (n=163, 61.5%) agreed that medical schools and residency training programs should develop policies to regulate the use of ChatGPT and AI by trainees. Moreover, the vast majority (n=165, 62.3%) recognized that ChatGPT’s answers required verification. When asked if their peers use ChatGPT ethically, 24.2% (n=64) of respondents disagreed and 54.7% (n=145) were unsure (Figure 1).

Gender differences in responses were noted. When compared to the female students, the male students were significantly more likely to believe that AI will improve diagnostic accuracy (n=47, 51.7% vs n=69, 39.7%; *P*=.001), reduce medical errors (n=53, 58.2% vs n=71, 40.8%; *P*=.002), and improve patient care (n=60, 65.9% vs n=95, 54.6%; *P*=.007).

After adjusting for gender, we found that there was no significant association between age and perceptions of AI in health care. As for previous ChatGPT experiences, after adjusting for age and gender, prior experience with ChatGPT in medical school was positively correlated with beliefs that AI will improve patient care, decrease medical error and misdiagnosis, and increase the accuracy of diagnoses (*P*=.001, *P*<.001, and *P*=.008, respectively).

**Table 1:** Participant demographic data (n=265).

Characteristic			Participants, n (%)
**Gender**			
	Female	174 (65.7)	
	Male	91 (34.3)	
**Age (years)**			
	20-24	138 (52.1)	
	25-30	110 (41.5)	
	31-35	17 (6.4)	
**Geographic region of medical school**			
	Africa	6 (2.3)	
	Asia	26 (9.8)	
	Europe	12 (4.5)	
	Middle East or North Africa	34 (12.8)	
	United Arab Emirates	187 (70.6)	
**Residency choice**			
	Anesthesia	4 (1.5)	
	Dermatology	17 (6.4)	
	Emergency medicine	23 (8.7)	
	Family medicine	13 (4.9)	
	Internal medicine or subspecialties	94 (35.5)	
	Obstetrics/gynecology	10 (3.8)	
	Pediatrics	40 (15.1)	
	Psychiatry	9 (3.4)	
	Radiology	16 (6)	
	Surgery or surgical specialties	37 (14)	
	Undeclared	2 (0.8)	

**Table 2:** Previous experiences with advanced technology or artificial intelligence (AI) and ChatGPT during medical school (n=265).

Survey questions		Participants, n (%)
**Advanced technology or AI**		
	Digital anatomy	163 (61.5)
	High fidelity simulation	96 (36.2)
	Virtual dissection	92 (34.7)
	AI-generated cases for simulation	71 (26.8)
	Computational pathology	70 (26.4)
**Tasks for which ChatGPT was used**		
	Complete written assignments	54 (20.4)
	Write case reports	44 (16.6)
	Write research papers	42 (15.8)
	Study or exam preparation	40 (15.1)
	Generate case scenarios	40 (15.1)
	Suggest research topics or questions	39 (14.7)
	Generate questions to test oneself	35 (13.2)
	Write patient notes	25 (9.4)

**Table 3:** Anticipated ChatGPT use during residency (n=265).

Survey questions	Participants, n (%)
Explore new medical topics or research	168 (63.4)
Study or exam preparation	151 (57)
Write research papers	127 (47.9)
Write case reports	122 (46)
Answer medical questions	115 (43.4)
Write patient notes	79 (29.8)
Clinical decision-making	73 (27.5)

## Discussion

### Principal Findings

In this cross-sectional study of 265 applicants to United Arab Emirates residency programs, most participants had minimal experience with ChatGPT in medical school but had positive perceptions of the technology and planned to use it during residency. Men and students with prior experience with the technology were significantly more likely to have positive views of the technology.

### AI Technology in Medical Education

Over the past decade, the use of AI and LLMs in health care has grown substantially in many areas. AI algorithms can provide clinical decision-making support and assist physicians in analyzing medical images, identifying high-risk patients, and recognizing potential drug interactions [24]. LLMs also have the potential to ease the burden of medical documentation by producing first drafts of patient progress notes, result notifications, and medical summaries, thereby saving valuable time that can be spent on patient interactions and tasks involving more advanced knowledge [25]. As the adoption of AI and LLMs continues to grow in health care, medical education must prepare learners for these developments. Integrating AI early in the medical curriculum will enable future physicians not only to be proficient users of AI tools, but to also take a leading role in steering, evaluating, and overseeing the technology to ensure its appropriate and ethical integration into training and clinical practice [26].

Our study adds to the AI literature by providing the perspectives of medical students—the end users of the technology. Our survey of medical trainees in a developed country in the Middle East shows minimal formal and informal experience with AI tools and limited perceptions of the potential uses of AI in health care. This appears to be a global problem. Our findings are consistent with other studies that show an inconsistent and fragmented approach to teaching AI and digital technology [27]. In a survey of US medical school students, 91.2% either denied or were unsure about their access to AI resources [28]. Further, a review of AI in undergraduate medical education found substantial variability and limited consensus on how to inculcate AI into the curriculum [26].

In our study, survey respondents displayed positive attitudes about the future of AI in education and medicine. However, less than half of the students anticipated integrating ChatGPT and AI tools into their studying or scientific writing. Previous experience was correlated with positive familiarity and perception, which is also supported by the literature [29]. LLMs such as ChatGPT can provide personalized learning with immediate, individualized feedback that can help trainees identify areas of weakness and improve performance [30]. These tools can also synthesize concepts from varied resources and can provide feedback on language and writing style [31]. For non–native English speaking students, this can defray the time and financial burdens of English language editing services and promote diversity and equity in the scientific publication landscape. Without proper guidance, our students may be missing out on these important opportunities.

### AI Technology in Medical Practice

Notably, only a small fraction of our study participants anticipated integrating AI technology into clinical practice; there was substantial ambiguity about the technology’s potential to improve decision-making. A recent Saudi study also showed that health care workers were highly interested in using AI tools for medical research (69.5%) but less so for decision-making (39.5%) or patient care (44.7%) [32]. Research has shown that LLMs can improve efficiency by completing routine tasks, such as writing discharge summaries or patient instructions [33,34]. Cascella et al [16] documented ChatGPT’s ability to create a medical note for a patient in the intensive care unit and correctly categorize treatments and test samples. This aligns with the observations made by Javaid et al [35] that ChatGPT can assist health care professionals with clerical tasks, including report creation and medical record transcription, which can streamline the clinical workflow and free up physician time to focus on patient care. The authors also observed that ChatGPT can be trained to match data from tests, laboratories, vital signs, and symptoms, and then provide recommendations [35]. Machine learning algorithms have also demonstrated the ability to improve diagnostic precision. In one study, an AI system was more successful than radiologists in interpreting medical images and predicting breast cancer [36]. Another study showed that AI had an accuracy rate of 71.7% in clinical decision-making [37]. Similarly, Liu et al [10] found that AI-generated suggestions could complement clinical decision-support alerts and assist experts in formulating their own recommendations.

Survey participants worried that AI would decrease the humanism in medical care. While some authors have argued that AI cannot provide the depth of awareness that human health care professionals have of the intricacies of medical care and the emotional and social circumstances of their patients [38], one study showed that in an online forum, chatbots generated high-quality responses to patient queries that were consistently rated to be more empathetic than physician responses [39].

It is notable that male survey participants had significantly more optimistic views of AI applications in health care than their female colleagues. There are several possible explanations for the observed gender differences in our study. Prior studies have shown that AI can perpetuate racial and gender biases and stereotypes [40]. Other research suggests AI might disproportionately benefit men in some domains, thereby widening the educational gender gap [41]. In addition, there are potential sociocultural factors, where due to traditional gender roles and societal expectations in the Middle East, men may have been exposed to AI and technology in a way that fosters more positive attitudes and leads to greater comfort and familiarity with these tools [42]. This can extend into the workforce, where women make up only 30% of those employed in the AI sector according to the 2023 World Economic Forum Global Gender Gap Report [43]. In response to these challenges, feminist AI has emerged as an approach to ensure that digital technologies are developed and used in ways that are equitable and inclusive [41]. While it is possible that these concerns contributed to the more tempered enthusiasm among female respondents, further research is needed to fully understand the underlying reasons.

Furthermore, we found no significant differences between age groups, likely because our cohort is young and of similar ages. In studies, younger, better educated, and more experienced individuals adopted AI technologies more readily [44]. We are reassured that the medical student respondents expressed some skepticism about the ethical impact of AI. Innovations like AI can have unintended consequences. Trainees can be encouraged to explore these tools under supervision and should be forewarned about potential issues of accuracy, reliability, bias, privacy, and academic integrity [45,46]. It is important for medical educators to reconcile the potential benefits and drawbacks of this disruptive innovation. To do this, the medical education community must develop core competencies in AI, as well as embed AI technology into clinical curricula and practice coupled with clear regulations on its use. Medical students and residents will also need AI ethics training to guide the responsible and equitable use of these technologies [12]. Some medical educators have already started this process. Suggested AI-related clinical competencies for health care professionals include basic knowledge of AI, social and ethical implications of AI, AI-enhanced clinical encounters that integrate diverse sources of information in creating patient-centered care plans, evidence-based evaluation of AI-based tools, and workflow analysis for AI-based tools [47]. Developing faculty expertise is an important first step in this process [46].

### Limitations

Our study has several limitations. Given the recent launch of ChatGPT, survey respondents had limited experience with this tool during medical school and their clinical rotations. Also, although respondents were from multiple medical schools in several countries, data collection was conducted at 1 hospital, limiting generalizability. Only graduating medical students were surveyed; understanding the experiences and perceptions of all medical trainees and teaching faculty is important. Finally, the cross-sectional design provides a snapshot view and does not capture long-term trends and changes in attitudes or use habits over time.

### Conclusions

ChatGPT and AI technology as a whole have the potential to revolutionize medical education and clinical practice. Our study shows that despite limited experience and some ethics concerns, medical students were overall positive and optimistic about the future of AI in medical education and health care but unclear about its role in their own training and careers. Structured curricula and formal policies and guidelines are needed to adequately prepare medical learners for the forthcoming integration of AI in medicine.

## Appendix

Multimedia Appendix 1 Checklist for Reporting Results of Internet E-Surveys (CHERRIES).

Multimedia Appendix 2 Questionnaire.

## Abbreviations

AI: artificial intelligence
CHERRIES: Checklist for Reporting Results of Internet E-Surveys
LLM: large language model
