# Supplementary material for: Medical Student Experiences and Perceptions of ChatGPT and Artificial Intelligence: Cross-Sectional Study
Source: JMIR Med Educ. 2023 Dec 22;9:e51302. doi: 10.2196/51302 (PMC10770787; doi:10.2196/51302)
Supplement: Multimedia Appendix 2 [file mededu_v9i1e51302_app2.docx]

**Multimedia Appendix 1: Questionnaire**


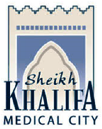
SKMC Research Ethics Committee

Survey Questions

1. **What is your gender?**

Male

Female

1. **What is your age?**

20-24

25-30

31-35

36-40

1. **In what region did you attend medical school?**

Africa (other)

Asia (other)

Australia/Oceania

Canada

Caribbean

Europe (other)

India

Ireland

Middle East/North Africa (other)

North America (other)

Pakistan

South America

United Arab Emirates

United Kingdom

United States of America

1. **What specialty are you pursuing in your residency (first choice)?**

Internal medicine

Family medicine

Emergency medicine

Radiology

Pediatrics

General Surgery

Obstetrics/gynecology

Psychiatry

Ophthalmology

Dermatology

Otolaryngology (ENT)

Neurology

Other, please specify

1. **In medical school, did you use ChatGPT for any of the following?**

- To assist with studying or exam preparation (yes/no)
- To generate questions to test yourself (yes/no)
- To generate case scenarios (yes/no)
- To suggest research topics or questions(yes/no)
- To help complete written assignments (yes/no)
- To help write research papers (yes/no)
- To help write case reports (yes/no)
- To help write patient notes (yes/no)

1. **In medical school, you used:**

- Digital anatomy (yes/no/I don’t know)
- Virtual dissection (yes/no/I don’t know)
- Computational pathology (yes/no/I don’t know)
- High fidelity simulation (yes/no/I don’t know)
- AI generated cases for simulation (yes/no/I don’t know)
- Virtual patients (yes/no/I don’t know)

1. **During residency, do you plan to use ChatGPT for the following?**

- To assist with studying or exam preparation (yes/no)
- To help answer medical questions (yes/no)
- To explore new medical topics or research (yes/no)
- To help write research papers (yes/no)
- To help write case reports (yes/no)
- To help write patient notes (yes/no)
- To assist in clinical decision making (yes/no)

1. **Please rate each of the following statements based and how much you agree with them:**

- ChatGPT/AI was effective in meeting my needs during medical school (strongly disagree/disagree/neutral/ agree/strongly agree)
- ChatGPT will improve my ability to learn during my residency (strongly disagree/disagree/neutral/ agree/strongly agree)
- I prefer using ChatGPT rather than Google or another search engine or medical reference to explain a medical topic (strongly disagree/disagree/neutral/ agree/strongly agree)
- The answers provided by ChatGPT need to be verified (strongly disagree/disagree/neutral/ agree/strongly agree)
- My peers have always used ChatGPT ethically (strongly disagree/disagree/neutral/ agree/strongly agree)
- Medical schools and residency programs should develop policies about the use of ChatGPT and AI by trainees (strongly disagree/disagree/neutral/ agree/strongly agree)

1. **Please rate each of the following statements based on how much you agree with them:**

- Artificial intelligence (AI) will have a major impact on healthcare during my career (strongly disagree/disagree/neutral/ agree/strongly agree)
- I am looking forward to using newer versions of ChatGPT and AI in my career (strongly disagree/disagree/neutral/ agree/strongly agree)
- AI will improve patient care during my career (strongly disagree/disagree/neutral/ agree/strongly agree)
- AI will decrease medical errors and misdiagnoses (strongly disagree/disagree/neutral/ agree/strongly agree)
- AI will enable me to make more accurate diagnoses as a physician (strongly disagree/disagree/neutral/ agree/strongly agree)
- AI will create more career opportunities for me as a physician (strongly disagree/disagree/neutral/ agree/strongly agree)
- AI’s potential has impacted my residency specialty choice (strongly disagree/disagree/neutral/ agree/strongly agree)
- I worry that AI will limit my job options in the future (strongly disagree/disagree/neutral/ agree/strongly agree)
- I worry that AI will reduce the humanistic aspect of medicine (strongly disagree/disagree/neutral/ agree/strongly agree)
- I worry that AI will reduce patient trust in physicians (strongly disagree/disagree/neutral/ agree/strongly agree)
- I worry about the ethical impact of AI on healthcare (strongly disagree/disagree/neutral/ agree/strongly agree)
